# Myocarditis in dogs: etiology, clinical and histopathological features (11 cases: 2007–2013)

**DOI:** 10.1186/s13620-014-0028-8

**Published:** 2014-12-24

**Authors:** Izabela Janus, Agnieszka Noszczyk-Nowak, Marcin Nowak, Alicja Cepiel, Rafał Ciaputa, Urszula Pasławska, Piotr Dzięgiel, Karolina Jabłońska

**Affiliations:** Division of Pathomorphology and Veterinary Forensics, Department of Pathology, Wroclaw University of Environmental and Life Sciences, Wroclaw, 50375 Poland; Department of Internal Medicine and Clinic of Diseases of Horses, Dogs and Cats Wroclaw University of Environmental and Life Sciences, Wroclaw, 50366 Poland; Department of Histology and Embryology, Wroclaw Medical University, Wroclaw, 50368 Poland; Department of Physiotherapy, University School of Physical Education, Wroclaw, 51612 Poland

**Keywords:** Myocarditis, Heart, Borrelia burgdorferi, Dog

## Abstract

**Background:**

Myocarditis is a disease caused by numerous etiological factors and characterized by a non-specific course. The only method allowing for precise characterization of inflammatory changes is the histopathological examination of heart muscle specimens. The study was conducted on heart muscle preparations from 11 dogs with ante-mortem diagnosis of cardiac disease. Animals presented with a poor response to an applied treatment or had suspected sudden cardiac death. The heart specimens were taken post-mortem, preserved and stained with haematoxylin and eosin. Subsequently, the presence and intensity of changes, i.e. inflammatory infiltration, the amount of connective tissue and features of cardiomyocyte degeneration were estimated. The specimens from dogs suspected of having a myocarditis of bacteriological etiology underwent additional bacteriological and immunohistochemical examination.

**Results:**

The examination revealed an inflammatory infiltration of variable intensity combined with the degenerative changes in all dogs. There were vegetative and abnormal cystic forms of *Borrelia burgdorferi* sensu lato in 6 dogs. A *Staphylococcus aureus* infection was confirmed in one dog and an acute coronary syndrome with neutrophil infiltration was revealed in another one.

**Conclusions:**

Although the clinical pattern in patients with myocarditis is diverse, the definitive morphological diagnosis is made based on the histopathological examination. This examination can lead to a better understanding of the pathogenesis of the disease. To the best of our knowledge, this is the first description of myocarditis combined with the presence of spore forms of *Borrelia burgdorferi* sensu lato in the heart specimens of dogs.

**Electronic supplementary material:**

The online version of this article (doi:10.1186/s13620-014-0028-8) contains supplementary material, which is available to authorized users.

## Background

Myocarditis is a heart disease rarely diagnosed in dogs. It can be caused by infectious agents such as bacteria, viruses and parasites. Depending on the aetiology, myocarditis can have various histopathologic patterns. It is usually non-specific and, although it is stated in the histopathologic examination, its direct cause can rarely be determined.

Causes of myocarditis in dogs include viruses (e.g. parvovirus, West Nile Virus), protozoal agents (i.e. Trypanosoma causing Chagas disease, Toxoplasma, Hepatozoon, Babesia), bacteria (i.e. *Staphylococcus, Streptococcus, Citrobacter, Bartonella, Borrelia*), fungal agents (i.e. *Coccidioides, Cryptococcus, Aspergillus*), helminths (Toxocara) and non-infectious factors such as autoimmune reactions, toxins, trauma, heat stroke and hemodynamic shock [1,2].

Infectious agents can cause acute or chronic changes through: (1) direct infiltration of inflammatory cells, (2) action of released toxins or (3) the delayed type immune response. The last one leads to a secondary inflammatory process as a result of the damage of cardiac muscle structure.

The clinical picture of myocarditis is diverse and can include both rhythm disturbances and at times changes resembling dilated cardiomyopathy [3].

Despite the development of serologic diagnostics and the possibility of identifying markers of myocardial damage [4], the only method that enables a reliable recognition of the type of cardiac muscle inflammation is an *ante mortem* cardiac biopsy or a histopathologic examination performed *post mortem* [5,6].

The purpose of this study was a histopathological analysis of 11 cases of myocarditis in dogs presenting with non-specific cardiac clinical symptoms that had a poor response to therapy, or suffered from sudden cardiac death.

## Methods

The study was carried out on *post mortem* samples from 11 dogs (7 males and 4 females) aged 2.5 to 13 years, weighing from 7 to 29 kg. The examined dogs included 5 cross-breed dogs, and one dog of each of the following breeds: German Shepherd, Miniature Schnauzer, Siberian Husky, Great Dane, Boxer and Cane Corso (Table 1).

**Table 1:** **Clinical findings, gross pathology and histopathological examination of studied dogs**

**Dog no.**	**Breed age sex**	**Clinical findings**	**Reason of death**	**Gross pathology**	**Histopathology and IHC**
1.	Miniature Schnauzer 3 y M	AF; signs of dilated cardiomyopathy; nephritis	Euthanasia due to heart failure	Generalized heart chamber dilation; ascites, hydrothorax, hydropericardium, enlargement of liver and spleen	Lympho-plasmocytic inflammation (+ to ++); cardiomyocyte degeneration (++ to +++); fibrosis (+ to ++); IHC: *B. burgdorferi* (+)
2.	German Shepherd 8 y M	AF; signs of dilated cardiomyopathy; recurrent lameness	Euthanasia due to heart failure	Generalized heart chamber dilation; thickening of pericardial sack; ascites, hydrothorax, hydropericardium, enlargement of liver and spleen	Lympho-plasmocytic inflammation (+ to ++); cardiomyocyte degeneration (+ to ++); fibrosis (+ to ++); IHC: *B. burgdorferi* (+)
3.	Siberian Husky 6 y F	VPCs; mass of unknown aetiology in LV	Sudden cardiac death	embolic material in aorta and LV	Granulocytic inflammation (+++); lympho-plasmocytic inflammation (+); cardiomyocyte degeneration (+++); IHC: *B. burgdorferi* (+)
4.	Mongrel 5 y F	no rhythm disturbances; signs of dilated cardiomyopathy	Euthanasia due to heart failure	Generalized heart chamber dilation; ascites, hydrothorax, hydropericardium, enlargement of liver and spleen	Lympho-plasmocytic inflammation (+); cardiomyocyte degeneration (+ to ++); fibrosis (+); IHC: *B. burgdorferi* (+)
5.	Mongrel 7 y M	no rhythm disturbances; signs of dilated cardiomyopathy	Euthanasia due to heart failure	Generalized heart chamber dilation; ascites, hydrothorax, hydropericardium, enlargement of liver and spleen	Lympho-plasmocytic inflammation (+ to ++); cardiomyocyte degeneration (+); fibrosis (+ to ++); IHC: *B. burgdorferi* (+)
6.	Mongrel 3 y F	no rhythm disturbances; signs of dilated cardiomyopathy	Euthanasia due to heart failure	Generalized heart chamber dilation; ascites, hydrothorax, hydropericardium, enlargement of liver and spleen	Lympho-plasmocytic inflammation (+); cardiomyocyte degeneration (+); fibrosis (+); IHC: *B. burgdorferi* (+)
7.	Mongrel 13 y M	VT with idioventricular rhythm; LVW hypertrophy and MR; dyspnoea	Euthanasia due to non-cardiac tumour	Hypertrophy of LVW and MV degeneration	Lympho-plasmocytic inflammation (+); cardiomyocyte degeneration (+);
8.	Cane Corso 3 y M	AF with rapid ventricular response; signs of dilated cardiomyopathy	Sudden cardiac death	Generalized heart chamber dilation	Lympho-plasmocytic inflammation (+ to ++); cardiomyocyte degeneration (++ to +++); fibrosis (+)
9.	Great Dane 2.5 y M	AF with rapid ventricular response; signs of dilated cardiomyopathy	Sudden cardiac death	Generalized heart chamber dilation	Lympho-plasmocytic inflammation (+ to +++); cardiomyocyte degeneration (+ to ++); fibrosis (+)
10.	Mongrel 4 y F	VT; LV dilatation; dyspnoea	Sudden cardiac death	Infarct of the LVW	Lympho-plasmocytic inflammation (+ to ++); granulocytic inflammation (+ to +++); cardiomyocyte degeneration (++ to +++)
11.	Boxer 7 y M	VT; no heart enlargement; neurological symptoms	Euthanasia due to neurological symptoms	No visible signs of heart failure	Lympho-plasmocytic inflammation (+ to ++); cardiomyocyte degeneration (+); fibrosis (+); lymphocytic inflammation of brain (+++)

AF atrial fibrillation, VPCs ventricular premature complexes, VT ventricular tachycardia, LV left ventricle, LVW left ventricular wall, MR mitral regurgitation, MV mitral valve, + − mild, ++ − moderate, +++ − severe, IHC immunohistochemistry.

### History, clinical examination and treatment

Intravitally, all dogs were symptomatic with clinical signs of heart disease that included exercise intolerance, cough or arrhythmia. Therefore, they underwent a clinical, electrocardiographic and echocardiographic examination. Patients showing rhythm disturbances underwent a 24-hour Holter ECG analysis. The ECG examination was performed using a BTL SD08® device (BTL, UK) with dogs in right lateral recumbency. The echocardiographic examination, performed in standard views using an Aloka SSD 4000® machine (Hitachi Medical Corporation, Japan), included: left atrium diameter to aorta ratio, end-systolic and end-diastolic left ventricular measurements, an assessment of the left ventricular shortening fraction and ejection fraction, blood flow velocity through the aortic and pulmonic valves and an estimation of the function of the atrioventricular valves. The 24-hour electrocardiographic (Holter) examination was performed using an Aspel ASPEKT 702® device (Aspel, Poland) compatible with the HolCARD computer system. Furthermore, all dogs underwent blood analysis including CBC, blood chemistry (ALT, AST, urea, crea, Na^+^, K^+^, Mg^2+^, Cl^−^ and cardiac troponin-I) and an antibody titer against *Borrelia sp*. A troponin-I level less than 0.07 ng/mL was considered normal as proposed by Sleeper et al. [7].

After identifying the heart disease, and depending on the diagnosis, the dogs underwent appropriate pharmacological treatment or an electrical cardioversion procedure. Six dogs with a positive antibody titer against *Borrelia sp*. received doxycycline (10 mg/kg *p.o.* for 28 days).

The dogs’ survival varied from 1 week to 5 months from the time of the diagnosis. Sudden cardiac death presumably caused by an arrhythmia (n = 3) or aorta embolism (n = 1) occurred in four dogs. Five dogs developed advanced heart failure, and were euthanized as per their owner’s decision. Two dogs were euthanized due to a concurrent non-cardiac disease.

### Post mortem examination

All dogs underwent a *post mortem* examination directly after death or euthanasia, which was performed due to a complex clinical picture, poor response to treatment or sudden cardiac death, and with the owners’ consent. According to Polish law, studies conducted on animal tissue do not require permission from the Ethical Board. Multiple heart specimens from the left ventricular free wall, right ventricular free wall, interventricular septum, left atrial wall and right atrial wall (including samples from sites showing macroscopical changes) were collected for further histopathologic examination. The specimens were fixed in 7% buffered formalin, embedded in paraffin blocks and sectioned at 6 μm. They were then stained using a standard H&E method, and subsequently underwent light microscopic evaluation at a 400× magnification. 20 photomicrographs of each studied specimen were subjected to computer-assisted image analysis, using a computer coupled to an optical Olympus BX53 microscope, equipped with an Olympus model Color View IIIa digital camera (Olympus, Japan). The specimens were analyzed for the presence and intensity of changes i.e. inflammatory infiltration, amount of connective tissue and features of cardiomyocyte degeneration using a semi quantitative scale (− no changes, + mild changes, ++ moderate changes, +++ severe changes). Specimens with an average count of inflammatory cells higher than 5 per field were recognized as positive for inflammatory infiltration [8]. An additional figure shows normal myocardial structure (see Additional file 1).

If granulocyte infiltration was present, further bacteriological examination, including a culture (blood agar and MacConkey agar), microscopic (Gram stain) and biochemical (API ID 32 Staf and 20 NE test) examination were performed.

Specimens from dogs with a positive antibody titer against *Borrelia sp.* underwent an immunohistochemical examination. The heart sections were deparaffinized in xylene and rehydrated by passing through a series of alcohol baths of decreasing concentration. The epitopes were unmasked in a citrate buffer (pH 6, 10 mM) at 96-98°C for 20 min. Endogenous peroxidase was blocked by incubating sections in 3% hydrogen peroxide. Non-specific binding sites were blocked using Antibody Diluent (30 min).

For detection of *Borrelia sp.*, specimens were incubated with the primary antibody diluted at 1:600 (polyclonal rabbit antibody; Serotec, cat. no 1439–9406, UK) for 1 h at room temperature. Goat secondary antibodies (EnVision™/HRP, Dako, Denmark) directed to rabbit immunoglobulins were bound to a dextran framework, conjugated with peroxidase. The immunoreaction was revealed by 3,3'-diaminobenzidine tetra-hydrochloride (DAB). The product of the reaction manifested itself as an intense brown colour and was located at the site of antigen presence.

According to the standard protocol, the final stages of the immunohistochemical reaction involved counterstaining with haematoxylin and a passage through a series of alcohol baths of increasing concentration. Finally, the preparations were coversliped with Euparal (Roth, Germany). Sections immunostained in the absence of a primary antibody were used as negative controls.

## Results and discussion

The results of clinical, *post mortem*, histopathological, and immunohistochemical examinations are shown in Table 1.

### Clinical symptoms, treatment and prognosis

The diagnosis was initially based on the clinical and diagnostic examination. The echocardiographic examination showed: heart chamber dilation, suggesting dilated cardiomyopathy in 7 dogs, dilation limited to the left ventricle in 1 dog, left ventricular wall hypertrophy with mitral regurgitation in one dog, a mass of unknown aetiology in the left ventricle of one dog and no changes in the heart of one dog.

Based on the ECG examination, 4 dogs were diagnosed with atrial fibrillation, 3 dogs with ventricular tachycardia, one dog with ventricular premature complexes and 3 dogs showed no rhythm disturbances.

Among 6 dogs showing a positive antibody titer against *Borrelia sp.*, one dog presented with nephritis diagnosed a few months earlier, and one dog exhibited recurrent lameness. In addition, one dog showed neurological symptoms suggesting a central nervous system disease.

Seven dogs had an elevated level of serum urea with normal creatinine level. 5 dogs had a slight elevation of the activity of hepatic enzymes, although these values did not exceed twice the upper limit of the normal range. No disturbances in electrolyte levels were noted. The value of the antibody titer against *Borrelia sp*. in 6 seropositive dogs varied from 1:64 to 1:256. All six dogs had an elevated level of cardiac troponin-I.

Acute myocardial inflammation in dogs is most frequently associated with various types of heart block, including 2^nd^ and 3^rd^ degree atrioventricular and sinus blocks [5,6,9]. None of the dogs in the described group demonstrated conduction blocks. Supraventricular and ventricular tachycardia, and changes imitating dilated cardiomyopathy described in our study and the presence of myocarditis are rarely interrelated [5,10-12].

The rhythm disturbances and impairment of heart function resulting from myocarditis often show a poor reaction to cardiologic treatment and lead to the death of the patient, such as that observed in the study group and confirmed by other authors [5,6,11].

According to Magnani et al. [11] and Mason et al. [13], the prognosis of human patients showing lymphocytic myocarditis is not affected by the type of treatment (including immunosuppressive drugs), though other research [10] suggests that anti-inflammatory treatment in patients with giant cell myocarditis can significantly prolong the survival time. Furthermore, in the case of acute lymphocytic myocarditis caused by *Borrelia burgdorferi*, the antibiotic treatment significantly improves the heart function, and limits the intensity of rhythm disturbances and heart failure symptoms [14-17]. The worsening of the clinical status and death despite the antibiotic treatment in dogs with borreliosis noted in our study may be a result of the chronicity of the process and secondary changes in the heart muscle structure, rather than the persistent presence of microorganisms. Goldstein et al. [18] noted a complete resolution of rhythm disturbances after an anti-inflammatory treatment in humans showing atrial flutter and fibrillation due to myocarditis resulting from invasive cardiologic procedures. A similar result could not be achieved by a standard antiarrhythmic treatment. The differences in the results found in literature could result from a varying etiology of the myocarditis (including viral) and various types of inflammatory infiltrates. However, this should not deter us from attempting to identify the underlying etiology and pathogenesis of the inflammatory processes.

### Post mortem examination

**Figure 1:**
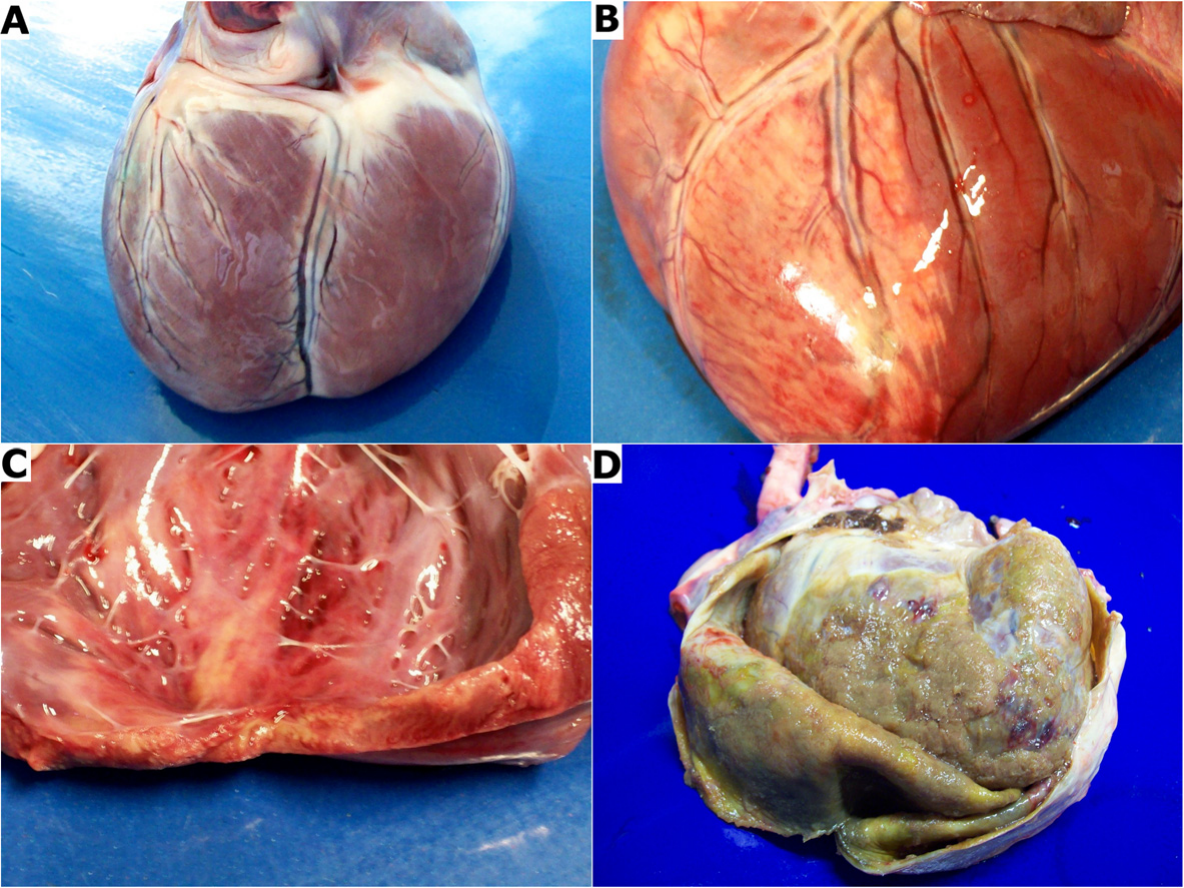
**The gross pathology. A** – generalized heart chamber dilation; **B** - infarct in the left ventricular wall; **C** – infarct site: changes in the heart muscle seen on cross-section; **D** – a thickening of pericardial sack with considerable amounts of fibrin covering the heart.

The *post mortem* examination confirmed results obtained via *ante mortem* echocardiographic examination. 7 dogs in our study had generalized cardiac chamber dilation (Figure 1A), 1 dog showed hypertrophy of the left ventricle wall with concurrent mitral valve degenerative changes, 1 dog had embolic material in the aorta and 1 dog had an infarct in the left ventricle wall (Figure 1B-C). Furthermore, 5 dogs presented with severe ascites, hydrothorax and hydropericardium with a concurrent enlargement of parenchymal organs and 1 dog had considerable thickening of the pericardial sac (Figure 1D).

The histopathological examination of the heart specimens revealed the presence of inflammatory infiltrates in heart muscle in at least one specimen from each of the dogs.

**Figure 2:**
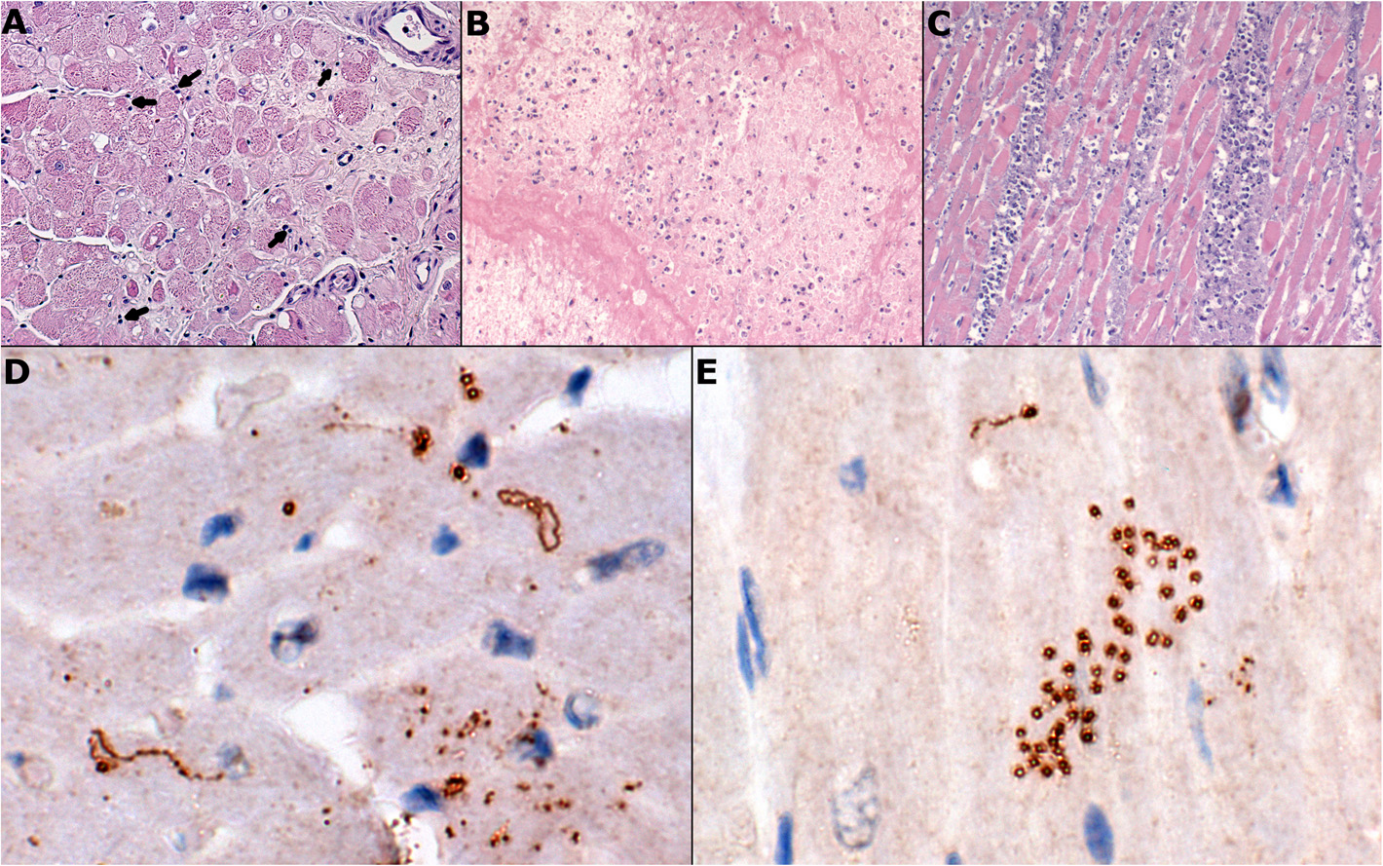
**Microscopic examination of heart specimens. A** - degeneration of myocardium with disturbances in cardiomyocyte structure, presence of fibrous tissue and mild inflammatory infiltrates (arrows) (H&E; 200×); **B** – severe cardiomyocyte degeneration accompanied by severe granulocytic and slight lympho-plasmocytic infiltration in heart tissue adjutant to embolic material (H&E; 200×); **C** – severe granulocytic and moderate lympho-plasmocytic infiltration of infarct site (H&E; 200×); **D** –vegetative and spore forms of *Borrelia burgdorferi* in heart specimens (IHC stain, 600×); **E** – *Borrelia burgdorferi* spore forms in heart specimens (IHC stain, 600×).

In 9 dogs, a diffuse lympho-plasmocytic infiltrate, more intense within the atrial than ventricular walls, was seen. The inflammatory cells were noted mainly subendocardially although they were also present throughout the myocardium. The inflammatory infiltrates were accompanied by foci of cardiomyocyte degeneration expressed by loss of striation, structural disturbances (e.g. swelling of the cardiomyocyte, blurring of cell structure and alterations in staining, Figure 2A), and the presence of abnormal nuclei (swollen nuclei with an altered structure and halo). In all study patients, the cardiomyocyte degeneration was more severe in the atrial walls than in the ventricular walls. Moreover, all the examined dogs were found to have a mild to moderate (from + to ++) fibrosis in the specimens from the atria. Furthermore, brain specimens from the dog showing neurological symptoms revealed severe lymphocytic inflammation.

In cases of conduction block in dogs, inflammation (mainly lymphocytic infiltrates) of variable intensity is situated within the heart conduction system [5,6,9]. When the inflammation occurs in other parts of the heart, the intensity of the infiltration in ventricles is weaker than in the atria [5,6,9]. This was also noted in the specimens we examined in this study. The lymphocytic infiltrates can be mild, moderate or severe. They are accompanied by an expansion of connective tissue and a loss of cardiomyocytes. The relationship between the intensity of inflammatory infiltrates and the incidence of cardiomyocyte degeneration is variable in every case and independent of the severity of the clinical signs [6]. The diffuse, mainly subendocardial character of the lympho-plasmocytic infiltrates stated in these 9 cases is likely to have resulted from the chronicity of the process.

In 1 dog, severe (+++) granulocytic infiltration was observed within the embolic material in the aorta and left ventricle, spreading to the left ventricular myocardium. This infiltration was accompanied by a mild (+) diffuse lympho-plasmacytic infiltration and foci of severe (+++) cardiomyocyte degeneration (Figure 2B). In the remaining slides from this dog, a diffuse lympho-plasmocytic infiltration (+) and mild (+) heart muscle degeneration were also noted. The bacteriological examination revealed the presence of *Staphylococcus aureus* within the embolic material and heart muscle.

In the cases of thromboembolic disease due to bacteria toxins, the infiltrates within the human heart tissue consisted of macrophages, T lymphocytes and neutrophils and were combined with cardiomyocyte degeneration. In general, the changes are more severe within the ventricles than atria [19]. The observed predominance of granulocytic infiltrates within the embolic material and adjoining parts of the left ventricle was probably due to an active bacterial infection.

The histopathological examination of one dog revealed a mixed lympho-plasmocytic (from + to ++) and granulocytic (from + to +++) infiltration within the site of the infarct and both atria (Figure 2C). There were no inflammatory cells in other slides evaluated from this dog. Simultaneously, in all of the heart specimens from the same dog a moderate to severe (from ++ to +++) cardiomyocyte degeneration was noted. The degenerative processes were more severe in the left than in the right side of the heart. There were no bacteria seen in the specimens collected from this dog. This patient showed the highest level of cardiac troponin-I (12.7 ng/mL).

Myocardial infarction is uncommonly reported in dogs. At the same time, it is a common disease in humans. increased levels of white blood cells (especially neutrophils) in the blood of patients after myocardial infarction are associated with a high risk of sudden cardiac death [20-22]. Furthermore, Distelmaier et al. [23] showed a higher mortality in humans with an accumulation of granulocytes within the culprit site compared to patients without them, though the role of neutrophils in the myocardial infarction is still unknown. In our study, we noted severe granulocytic infiltrates mainly within the infarct site combined with a rapid course of disease and sudden cardiac death.

Furthermore, in slides from 6 dogs showing a positive antibody titer against *Borrelia sp.* (including the dog with aortic embolism), the presence of *Borrelia burgdorferi* sensu lato and the presence of abnormal cystic forms, considered to be spores of the spirochetes, were confirmed by immunohistochemistry (Figure 2D-E). The cardiac troponin-I levels ranged in these dogs from 0.25 ng/mL to 1.7 ng/mL. Borreliosis is very common in both humans and animals in northern America, Asia and Europe, including Ireland [24,25]. Reports suggest the disease is spreading to new regions and should therefore be taken into account during the diagnostic process even in non-endemic regions [26,27]. Myocarditis secondary to borreliosis is rarely reported in dogs. Agudelo et al. [28] described a case of a Boxer dog suspected of heart borreliosis with cardiomegaly, atrial fibrillation and heart failure symptoms. However, the authors did not perform a histopathologic examination of the heart to confirm the presence of spirochetes or inflammatory infiltrates. The influence of *Borrelia burgdorferi* sensu lato on the heart muscle in both humans and dogs is most often seen in the early phase of the disease (approximately 3 weeks after the infection. Yet, it can also be noted in the chronic phase [16,29]. In the course of heart borreliosis, the symptoms described most often are atrioventricular blocks although other rhythm disturbances and signs of heart failure are also observed [14-17,29,30]. In the acute phase of the disease, the histopathological examination most frequently reveals an intense acute transmural interstitial lympho-plasmocytic infiltration [17]. With time, the number of spirochetes in the heart muscle decreases, which is also accompanied by the reduction in the severity of inflammatory infiltrates that become diffusely spread in the myocardium [14]. It is very difficult to demonstrate spirochetes in heart specimens [14]. In our study, the visualisation of borrelias was not possible without using a specific immunohistochemical stain.

In people with typical Lyme disease no cardiomyocyte degeneration is observed and the level of heart enzymes remains within the normal range. In experimentally infected dogs, a similar histopathological pattern was observed in the acute phase of the disease [14]. The presence of variable amounts of myocardial degeneration and more than 5 times the upper end of normal elevated levels for troponin-I in the described cases may be a result of the chronicity of the process. There is also a hypothesis that the destructive influence of *Borrelia sp.* on the heart muscle is not a direct result of microorganism presence, but of toxins produced by spirochetes [14]. In such a case, the antibiotic treatment, causing death and destruction of the bacteria, would result in the release of the toxins and an inflammatory response of the heart muscle [14]. It is also suspected that the inflammation in the chronic phase of the disease can result from an autoimmune reaction caused by the presence of the spirochetes and not of the microorganisms themselves [14,16].

The authors did not find reports describing the presence of spore forms of *B. burgdorferi* sensu lato in the heart muscle in dogs. Miklossy et al. [31] described forms similar to the ones shown in our paper in cases of chronic nervous system borreliosis in humans.

In none of the specimens from dogs showing heart chamber dilation were there attenuated wavy fibres comprising at least half of the thickness of the ventricular myocardium (as a specific histopathological feature for dilated cardiomyopathy [32]). This, together with the presence of inflammatory infiltrates, led to the diagnosis of secondary dilated cardiomyopathy.

It was not possible to determine the direct cause of lympho-plasmocytic infiltrates within the heart muscle in three dogs. Often, despite the broad spectrum of tests, it is impossible to define the cause of myocarditis [5,6,33]. Nonetheless, nonspecific clinical signs of myocardial inflammation should trigger a histopathological examination, especially in cases refractory to treatment, to decrease the group of so-called idiopathic heart diseases. After performing heart biopsies in 50 patients with idiopathic atrioventricular blocks, Uemura et al. [34] found that the presence of myocarditis resulted in the development of arrhythmia in 6% of cases.

## Conclusions

Dogs showing nonspecific clinical signs, poor response to treatment or sudden cardiac death should have a *post mortem* examination as often as possible. Such an examination would help to advance our understanding of the mechanisms of internal and infectious diseases influencing the heart muscle. Based on the histopathological examination, a definitive morphological diagnosis of myocarditis can be made. An immunohistochemical examination allows one to visualise the presence of vegetative and spore forms of *B. burgdorferi* sensu lato in tissue specimens in the course of heart borreliosis.

## Additional file

Additional file 1
**Normal myocardial structure with slight amount of interstital connective tissue and no cardiomyocyte degeneration (H&E stain).** A - right ventricular myocardium; B - left ventricular myocardium.
