# Supplementary figures and images for: Myocarditis in dogs: etiology, clinical and histopathological features (11 cases: 2007–2013)
Source: Ir Vet J. 2014 Dec 24;67(1):28. doi: 10.1186/s13620-014-0028-8 (PMC4311452; doi:10.1186/s13620-014-0028-8)

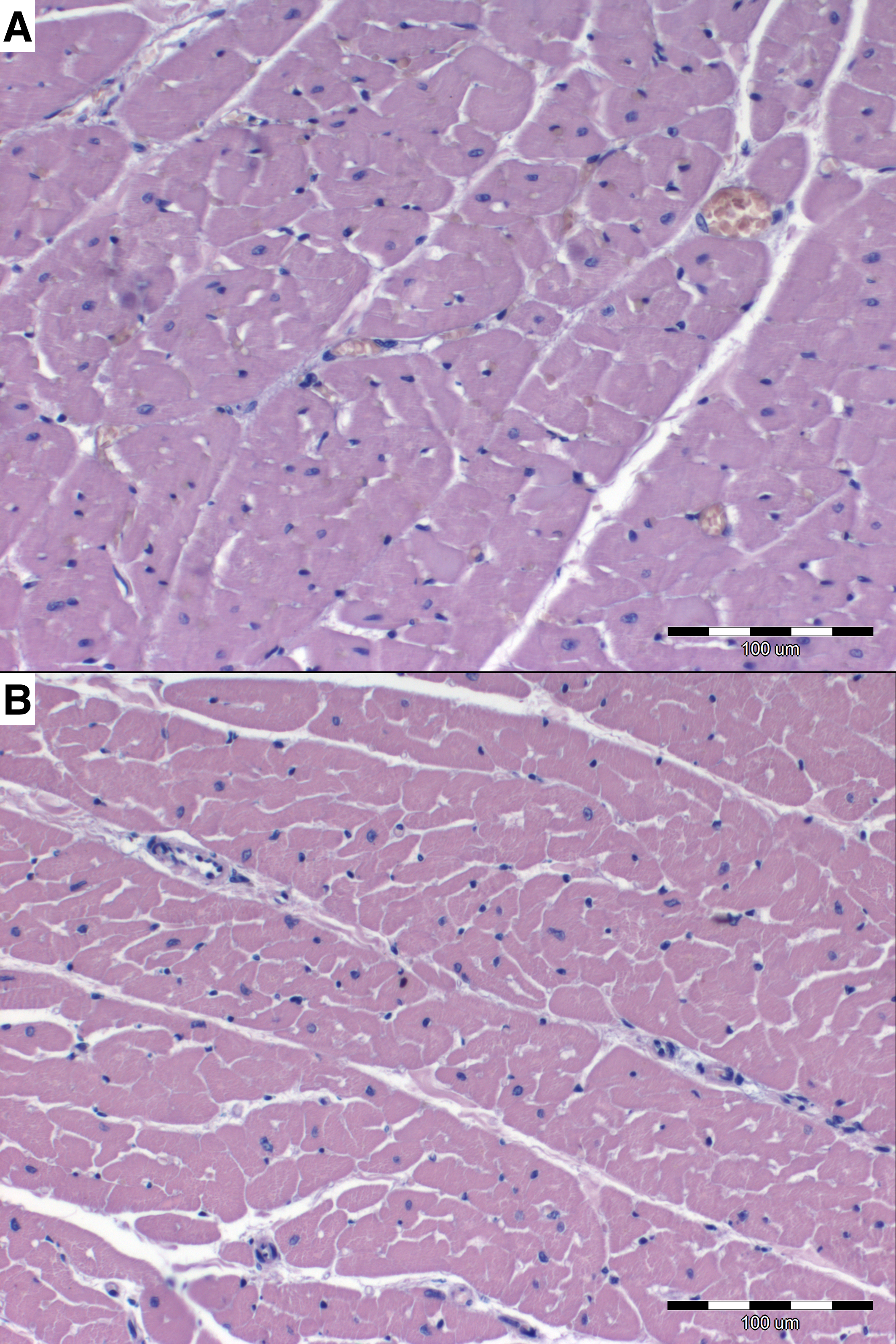

Supplement: Additional file 1 — Normal myocardial structure with slight amount of interstital connective tissue and no cardiomyocyte degeneration (H&E stain). A - right ventricular myocardium; B - left ventricular myocardium. [file 13620_2014_28_MOESM1_ESM.jpg]
